# A Rare Presentation of Colorectal Cancer with Unusual Progressive Intramuscular and Subcutaneous Metastatic Spread

**DOI:** 10.22038/AOJNMB.2018.11934

**Published:** 2019

**Authors:** Reyhaneh Manafi-Farid, Narjess Ayati, Mohammad Eftekhari, Babak Fallahi, Fardad Masoumi

**Affiliations:** 1 Research Institute for Nuclear Medicine, Shariati Hospital, Tehran University of Medical Sciences, Tehran, Iran; 2 Nuclear Medicine Research Center, Mashhad University of Medical Sciences, Mashhad, Iran; 3 Tabriz University of Medical Sciences, Tabriz, Iran

**Keywords:** Colorectal carcinoma, Muscle metastasis, Rare metastasis, ^18^F-fluorodeoxyglucose PET/CT

## Abstract

Colorectal carcinoma is one of the most common causes of cancer-related death, worldwide. Recently, due to the introduction of novel imaging and therapeutic techniques, five-year survival of patients has increased. However, distant metastasis is still expected in half of the patients. Colorectal cancer tends to target the abdominal cavity, liver, lungs, and bones as the common sites of metastasis. Nevertheless, rare cases of muscle metastasis have been reported. This report presents a 23-year-old male, who despite chemotherapy, demonstrated gradual progressive disease and metastases to the submandibular region, lungs, adrenal gland as well as muscles and subcutaneous tissues. He had developed multiple asymptomatic muscular metastases metachronously over two-year time period discovered on an ^18^FDG-PET/CT, namely in the deltoid, external oblique abdominis, rectus abdominis, and quadriceps muscles, as well as one of the extrinsic muscles of the tongue. The presence of distant, especially extrahepatic metastasis, adversely affects the prognosis of colon carcinoma. Since limited cases of muscle metastasis have been reported in carcinoma of colon, the underlying pathophysiology, optimum treatment, and prognostic issues are yet to be substantiated.

## Introduction

Colorectal cancer (CRC) is the second and the third most diagnosed malignancy (excluding non-melanoma skin cancers) in females and males, respectively (1). Additionally, it is the third leading cause of cancer-related death in developed countries (1) resulting in related mortality in 50% of the patients (1). A reduction in mortality rate has been observed in recent years which is due to better screening protocols, high-tech treatment procedures, new chemotherapeutic medications , and precise follow-up visits along with reducing predisposing risk factors in the developed areas (1, 2). With the emergence of recent sophisticated diagnostic and therapeutic modalities, 5-year-survival of CRC has reached 60% (3). Keeping in mind that the metastasis is a common manifestation in CRC, the rate of 25% metastatic disease in the initial diagnosis is not beyond expectation (4). It has been reported that 50% of patients will develop metastasis during their lifetime struggling with cancer (3). It has been proved that surgical procedures and removal of liver metastases have augmented the 5-year survival of cases suffering from metastatic colorectal cancer (mCRC) up to 25-40% (5). Fortunately, mean survival for mCRC has improved more than 200%, approaching 30 months in the last 20 years (2).

Various methods, such as ultrasound, computed tomography, magnetic resonance imaging, and ^18^FDG-PET, are employed to discover metastases in CRC. It is a well-established fact that ^18^FDG-PET is an effective modality in the evaluation of metastasis in CRC, especially when CEA (carcinoembryonic antigen) level is rising, and no distinct anatomic evidence of tumoral lesion could be identified. In the meantime, ^18^FDG-PET is more reliable in detecting extrahepatic or extrapulmonary metastases (3).

The incidence of diagnosis of the extrahepatic metastasis (EHM) is reported to be approximately 32% using ^18^FDG-PET (4). ^18^FDG-PET is believed to decrease not only dispensable laparotomies in 50% of cases (6) but also the number of non-curative metastasectomy procedures (4). On the other hand, the benefits of performing ^18^FDG-PET for all CRC patients is yet to be affirmed (2). The most common organs involved by metastases from CRC are liver, lung, and peritoneum (7). 

The prognosis of mCRC deteriorates with the presence of EHM, which interferes with the possible chance of curative surgery. Hence, 5-year survival drops to 26% (6).

## Case report

**Figure 1:**
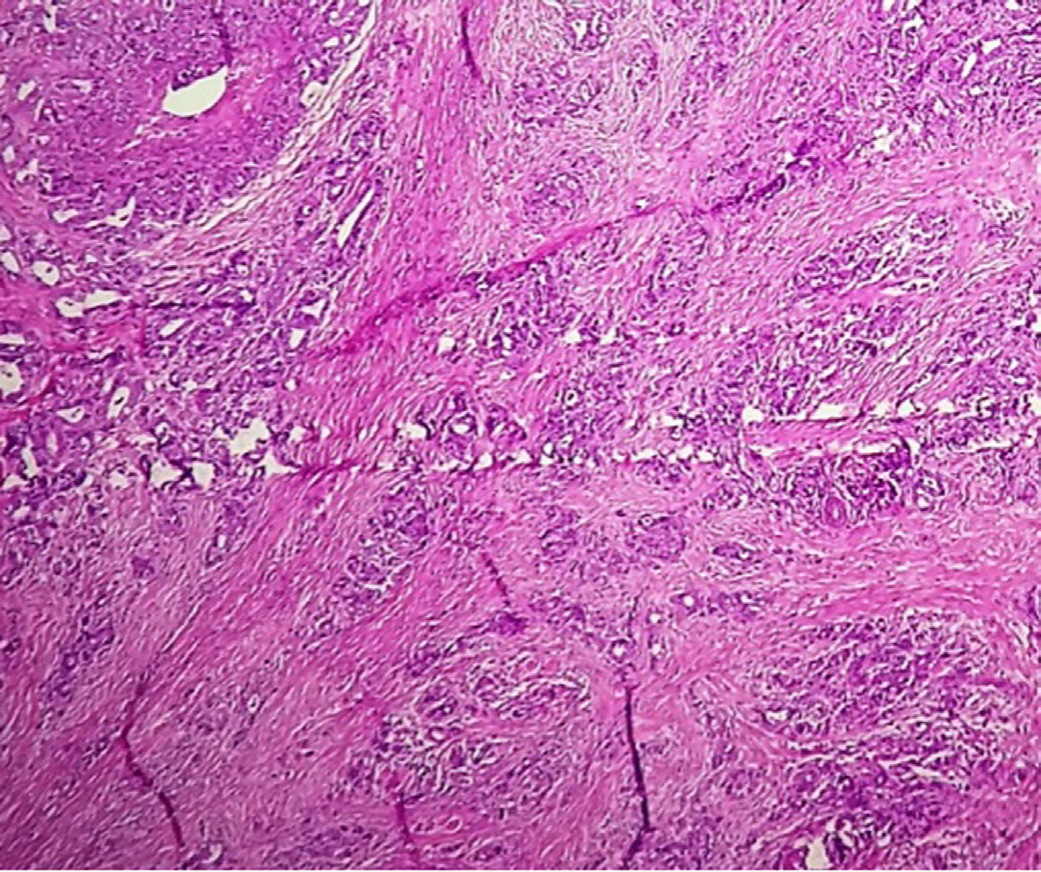
Histological appearance of the primary sigmoid carcinoma revealing infiltration of malignant cells into all layers of the intestinal wall

A 19-year-old male was evaluated for painless hematochezia. A rectal mass was detected, measuring 3×2×1.5 cm. The histopathology of the tumor was compatible with infiltrative, ulcerative adenocarcinoma with the mucin-producing features, involving full intestinal wall thickness as well as a corresponding mesocolic lymph node (pT3N1Mx), KRAS and NRAS wild-type (Figure 1). Hence, the patient received neoadjuvant chemotherapy, underwent proctocolectomy and subsequently adjuvant chemotherapy, including the FOLFOX regimen in 2014.

**Figure 2:**
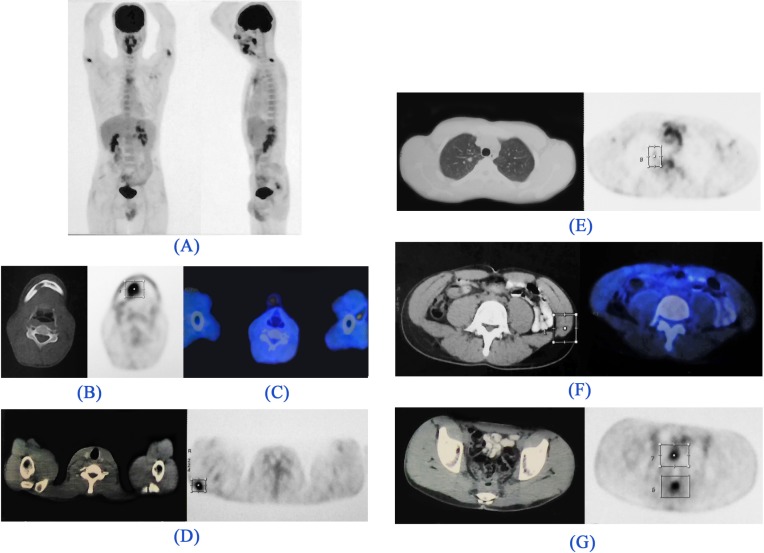
^18^FDG-PET/CT images. *(A). Maximum intensity projection (MIP) image:* There are foci of metabolically active lesions in the right paramedian submandibular region, bilateral proximal upper extremities, and posterior pelvic cavity. Metabolically active lesions are demonstrated in the *(B). Trans-axial CT (left) and PET (right) images:* Right submandibular region (right external lingual muscles) with invasion to the adjacent mandible, *(C). Trans-axial Fused image:* Posterior to the left biceps muscle, *(D). Trans-axial CT (left) and PET (right) images:* Right deltoid muscle, *(E).* *Trans-axial CT (left) and PET (right) images:* Pulmonary nodule in the apical segment of the right lung, *(F).* *Trans-axial CT (left) and Fused (right) images:* Left external oblique abdominis muscle, *(G).* *Trans-axial CT (left) and PET (right) images:* GI tract, above the anastomotic region (which was proved to be non-malignant in subsequent colonoscopic and biopsic evaluation).

**Figure 3:**
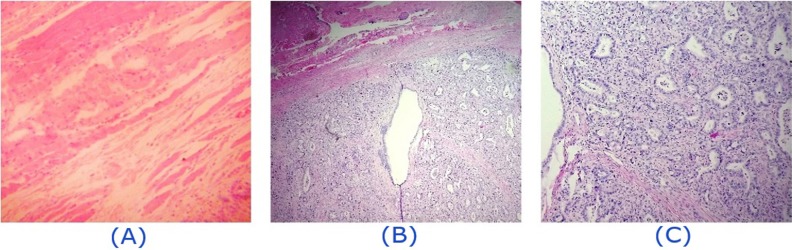
Histopathologic findings of the hypermetabolic deltoid mass showing atypical glands lined by malignant cells concomitant with inflamed fibrotic stroma suggestive of muscle metastasis from the colon cancer. *(A):* Skeletal muscle fibers surrounded by inflammatory cells *(B):* Atypical glandular tissue with muscular fascicles in the top of the field. *(C):* Atypical glands lined by malignant cells

**Figure 4:**
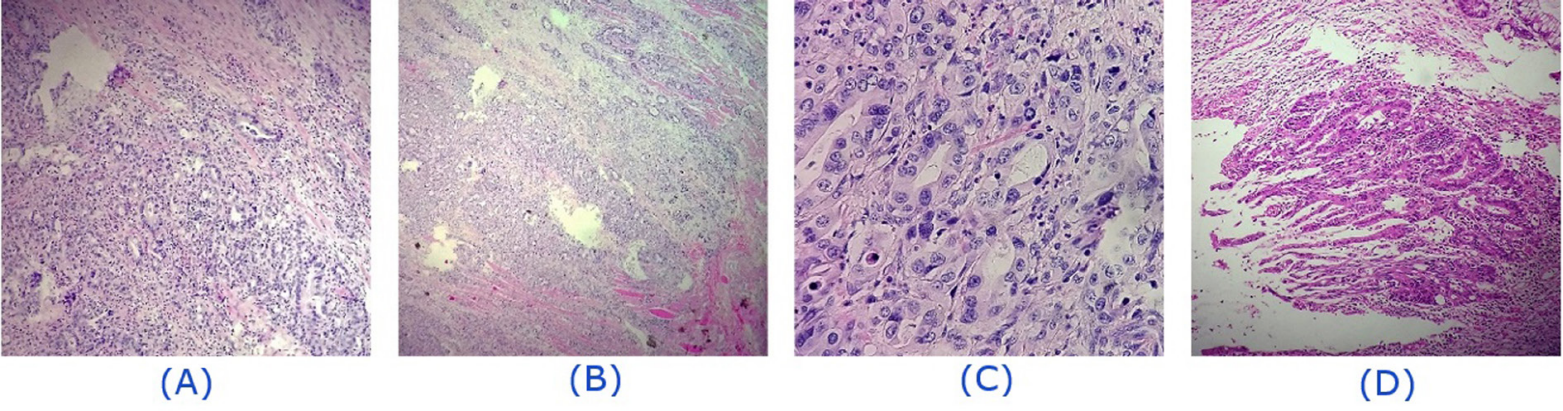
Histopathologic findings of the hypermetabolic tumoral mass in the right chin, involving muscular and bone structures. Fibrocollagenous and skeletal muscle tissue are infiltrated by proliferating atypical neoplastic cells showing glandular differentiation. *(A, B):* Fibrocollagenous and muscular tissues infiltrated by atypical cells, glandular structures as well as inflammatory cells. *(C):* Glandular differentiation. *(D): *Atypical glandular structure formation

**Figure 5:**
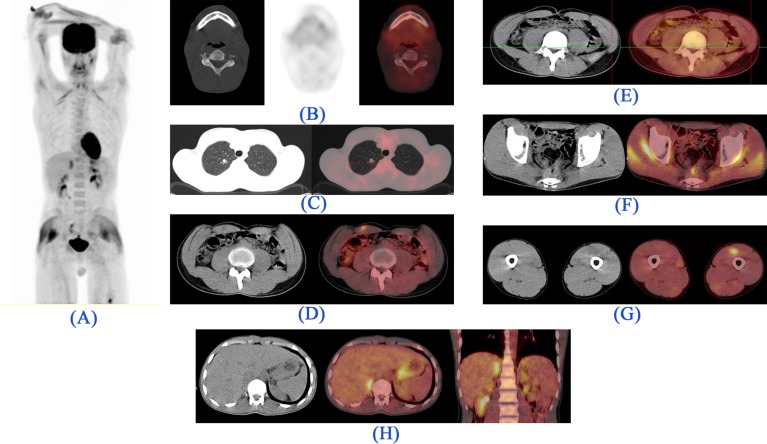
^18^FDG-PET/CT images. *(A). Maximum intensity projection (MIP): *Foci of metabolically active lesions are visualized superior to the right kidney, right mid abdominal region, and the posterior pelvic cavity. In addition, diffuse metabolic activity is noted in skeletal muscles owing to physical activity in the morning of the study. Of note, due to technical issues the imaging of the lower extremities was performed separately. *(B). Trans-axial CT (left), PET (middle) and Fused (right) images:* Comparing with the previous study, the mandibular bone lesion is healed without evidence of remaining tumoral lesion. *(C).* *Trans-axial CT (left) and Fused (right) images*: A mild metabolically active pulmonary nodule in the apical segment of the right lung shows stable size and metabolic activity. *(D).* *Trans-axial CT (left) and Fused (right) images*: A new metabolically active lesion in the right rectus abdominis muscle. *(E).* *Trans-axial CT (left) and Fused (right) images*: A metabolically inactive lesion in the left external oblique abdominis muscle probably reveals the metabolic response to the treatment. *(F).* *Trans-axial CT (left) and Fused (right) images*: Mild metabolic activity in the GI tract, above the anastomotic region, reveals no significant change in size and metabolic activity in comparison to the previous study (which has been proven to be non-malignant in recent colonoscopic and biopsic evaluation). *(G).* *Trans-axial CT (left) and Fused (right) images*: A new metabolically active metastasis in the left quadriceps muscle. *(H).* *Trans-axial CT (left), Fused (middle), and Coronal Fused (right) images*: A new metabolically active metastatic lesion in the right adrenal gland

During the course of chemotherapy, the patient developed a painless right submandibular mass, ignored by him. Chemotherapy sessions continued until May 2016, when he had his first ^18^FDG-PET/CT, for evaluation of response to treatments as well as the new emerging pain in his chin. Unexpectedly, several ^18^FDG-avid foci were discovered in the right deltoid, left external oblique, posterior left biceps brachii muscles, as well as a hypermetabolic soft-tissue mass in the region of the right external tongue muscle accompanied by a lytic right mandibular lesion, suggesting metastatic disease. 

In addition, a 9 mm pulmonary nodule revealing modest metabolic activity was detected in the apex of the right upper lobe, highly suggestive of metastasis (Figure 2). Confirmatory excisional surgery was carried out on the right deltoid lesion (Figure 3), the most hypermetabolic muscular metastasis, as well as the right submandibular mass (Figure 4). As expected, these lesions were proved to be metastatic adenocarcinoma. 

The other lesions did not undergo any further evaluation since these pathologically proven metastatic lesions were convincing enough to commence additional chemotherapy courses, the FOLFIRI regimen.

Immediately after termination of the chemotherapy, another painless lesion emerged on the scalp, which was proved to be subcutaneous metastatic adenocarcinoma on biopsy. However, this time, the patient refused to undergo any further treatment. 

After three months, another lesion became apparent in the occipital scalp bringing about discomfort during sleep. Afterwards, the patient was reevaluated by ^18^FDG-PET/CT at the end of 2017 (Figure 5). The imaging revealed foci of metabolic activity in the right rectus abdominis and left quadriceps muscles. However, the occipital lesion, measuring 1.5×1.5 cm, showed no abnormal FDG uptake. At this time an additional hypermetabolic focus consistent with metastasis was detected in the right adrenal gland.

## Discussion

This case is an example of an uncommon presentation of colorectal cancer demonstrating a solitary pulmonary nodule in the presence of multiple progressive muscular and soft tissue tumoral metastatic lesions sparing the liver, the most common target organ for metastasis in CRC (5) which is an extremely rare manifestation in this malignancy. 

While the skeletal muscles make up the bulk of body mass and receive substantial blood flow, intramuscular metastasis is a rare phenomenon in all types of cancers. However, with the emergence of new imaging methods, such as MRI and ^18^FDG-PET/CT, muscle metastases are detected in growing numbers (8). Muscular metastasis of CRC could be presented as a severely painful mass or asymptomatic lesions, incidentally detected in ^18^FDG-PET studies (9). 

According to Hasegawa et al., one plausible reason for the rarity of muscular metastasis can be explained by active movements of the muscles and turbulent blood flow through vessels during exercise making the environment remarkably inappropriate for implantation of the tumoral cells (8). 

In addition, Hasegawa et al. holds that metabolism products in exercising muscles, such as lactate, create an acidic environment prohibiting tumoral cells from proliferation (8). The presented patient, suffering from multiple muscular metastases, was an athlete, who was doing a considerable amount of exercise. Thus, this theory cannot adequately explain the infrequency of metastasis, especially in our patient.

CRC, similar to other types of cancer, possesses a wide range of heterogeneity showing various growth patterns even in a single tumor. These morphological diversities are a consequence of either genetic or non-genetic factors (10). It has been documented that the presence of particular gene mutations, such as *RAS* (RAS proto-oncogene, *GTPase*), *BRAF* (B-Raf proto-oncogene, serine/threonine kinase), and *MSI* (microsatellite instable) indicates a poorer prognosis (11). In addition, gene mutations extensively affect the presentation of the tumor, its progression, and even therapeutic approach (11). *KRAS* and *BRAF* mutation, for instance, lead to more lung and early peritoneal metastases, respectively (10, 11). Further than genes themselves, epigenetic regulations, post-translational modifications of genes, as well as tumoral microenvironment have been demonstrated to impact the morphological diversities (10). Of note, the mucin-producing CRC has been shown to demonstrate more *BRAF* mutation, extrahepatic metastasis, inadequate response to treatment, and poorer prognosis (12). 

It is evident that extrahepatic disease implicates poorer prognosis and reduces the survival. However, due to the limited numbers of documented muscle metastases and, more importantly, concomitance with other sites of metastasis throughout the body, the sheer impact of this expression in the reduction of life expectancy is not ascertainable by the existing data. Considering substantial under detection of asymptomatic muscular metastasis, we theorize that the prognosis of this phenomenon may not be as poor as it is reckoned. To give an example, our patient is still alive, approximately two years after diagnosis of the first muscle metastasis, despite his refusal to undergo further treatment. 

Finally, it is of note that the adrenal metastasis, which is also observed in our patient, is another relatively rare manifestation of CRC. The rate of metastasis to adrenal glands is estimated 8.6% to 27.0% in all primary malignant carcinomas as opposed to 1.9% to 17.4% for CRC in the literature (13), an interesting observation worthy of being studied separately.

In summary, muscle metastasis is an uncommon presentation of colorectal carcinoma, alarming systemic disease spread, which in turn, aggravates the patient’s condition. We present a young patient, suffering from colorectal carcinoma, with multiple metastases to muscles, soft-tissues, lung and adrenal gland discovered on ^18^FDG-PET/CT. 
